# Dorsal anterior cingulate cortex modulates supplementary motor area in coordinated unimanual motor behavior

**DOI:** 10.3389/fnhum.2015.00309

**Published:** 2015-06-04

**Authors:** Avisa Asemi, Karthik Ramaseshan, Ashley Burgess, Vaibhav A. Diwadkar, Steven L. Bressler

**Affiliations:** ^1^Center for Complex Systems and Brain Sciences, Florida Atlantic UniversityBoca Raton, FL, USA; ^2^Brain Imaging Research Division, Department of Psychiatry and Behavioral Neuroscience, Wayne State University School of MedicineDetroit, MI, USA; ^3^Department of Psychology, Florida Atlantic UniversityBoca Raton, FL, USA

**Keywords:** dorsal anterior cingulate cortex (dACC), supplementary motor area (SMA), primary motor cortex (M1), motor control, connectivity analysis, adolescence

## Abstract

Motor control is integral to all types of human behavior, and the dorsal Anterior Cingulate Cortex (dACC) is thought to play an important role in the brain network underlying motor control. Yet the role of the dACC in motor control is under-characterized. Here we aimed to characterize the dACC’s role in adolescent brain network interactions during a simple motor control task involving visually coordinated unimanual finger movements. Network interactions were assessed using both undirected and directed functional connectivity analysis of functional Magnetic Resonance Imaging (fMRI) Blood-Oxygen-Level-Dependent (BOLD) signals, comparing the task with a rest condition. The relation between the dACC and Supplementary Motor Area (SMA) was compared to that between the dACC and Primary Motor Cortex (M1). The directed signal from dACC to SMA was significantly elevated during motor control in the task. By contrast, the directed signal from SMA to dACC, both directed signals between dACC and M1, and the undirected functional connections of dACC with SMA and M1, all did not differ between task and rest. Undirected coupling of dACC with both SMA and dACC, and only the dACC-to-SMA directed signal, were significantly greater for a proactive than a reactive task condition, suggesting that dACC plays a role in motor control by maintaining stimulus timing expectancy. Overall, these results suggest that the dACC selectively modulates the SMA during visually coordinated unimanual behavior in adolescence. The role of the dACC as an important brain area for the mediation of task-related motor control may be in place in adolescence, continuing into adulthood. The task and analytic approach described here should be extended to the study of healthy adults to examine network profiles of the dACC during basic motor behavior.

## Introduction

Executive control is a central organizing principal underlying human brain network interactions and a ubiquitous element of higher human function (Royall et al., 2002; Banich, 2009). Motor control is an important and specific form of control that subserves multiple complex behaviors, especially the integration of motor responses with cognitive decisions (Paus, 2001). The dorsal anterior cingulate cortex (dACC) has been strongly implicated in adult motor control. Tasks requiring motor control (including both the inhibition and excitation of motor responses) consistently demonstrate robust activation of the adult human dACC.

Cognitive-control-related brain networks, typically studied using complex response inhibition tasks, appear to be functionally articulated by the time of adolescence (Mennigen et al., 2014). Although the dACC continues to develop into the second decade of life (Gogtay et al., 2004), its role in mediating basic motor control in adolescence has been relatively under-studied. Our goal here was to investigate network interactions of the adolescent dACC: its undirected and directed interactions with the supplementary motor area (SMA), a high-level constituent of the motor system that has been associated with the control of simple finger movements, and with the primary motor cortex (M1). We attempted this using a simple unimanual visuomotor integration task with basic motor control demands. The task is highly suitable for characterizing brain network effects in adolescence as it is readily performed with no developmental changes in performance. Moreover, evidence of systematic network effects of the dACC in adolescence provides suggestive evidence for the early establishment of brain network interactions subserving this basic domain in the adult. Our results indicate that the adolescent dACC undergoes task-related undirected functional coupling with both the SMA and M1, but sends task-specific directed signals specifically to the SMA. These results suggest hierarchical control-related interactions between the dACC and SMA as part of a large-scale executive brain network underlying simple motor control in adolescence that most likely continues into adulthood.

The anterior cingulate cortex is a structurally and functionally complex frontal region (Bozkurt et al., 2005; Margulies et al., 2007). It has been proposed that the dorsal part of the anterior cingulate cortex (dACC, also called the anterior midcingulate cortex) participates in the control of behavior by acting as a major interface between sensorimotor and cognitive processing (Paus, 2001). The anterior cingulate cortex is perhaps best known as a mediator of cognitive conflict (Botvinick et al., 1999; Cohen et al., 2000, 2005; Milham and Banich, 2005; Aarts et al., 2008; Brown, 2009). However, the dACC is connected with the frontal motor system in both humans (Barbas, 1986; Luppino et al., 1990, 1991; Paus et al., 1993; Koski and Paus, 2000; Schulz et al., 2011; Amiez and Petrides, 2014) and non-human primates (Matelli et al., 1991; Morecraft and Van Hoesen, 1992; Vogt et al., 1992, 2005; Devinsky et al., 1995; Picard and Strick, 1996, 2001; Dum and Strick, 2002; Morecraft et al., 2012). That the dACC is also involved in motor function is seen by evidence from experimental and clinical lesion, electrical stimulation, and PET studies (Paus, 2001). In monkeys, unilateral cingulate lesions produce contralateral motor neglect (Watson et al., 1973). In humans, excessive activity in the dACC in the cingulate epilepsy syndrome is associated with motor impairment (Mesulam, 1981; Devinsky et al., 1995). Other human neurological syndromes that impact the dACC also involve disruption of motor control or cognitive processes associated with motor events (Devinsky et al., 1995; Paus, 2001). Electrical stimulation of the dACC elicits simple and complex contralateral and bilateral movements in both monkeys and humans (Luppino et al., 1991). PET studies confirm that motor regions of the human dACC are activated during manual movement (Paus et al., 1993, 1998). The dACC remains neurodevelopmentally dynamic in adolescence (Fjell et al., 2012) though this does not preclude highly articulated interactions with other brain regions, in relatively simple sensori-motor domains such as basic motor function (Ordaz et al., 2013).

The dACC is ideally suited to serve as a motor control area of both the adolescent and adult human brain: its dense projections to various regions of motor cortex suggest that the structure is in a privileged topological position to modulate their activities. Nonetheless, relatively few functional Magnetic Resonance Imaging (fMRI) studies have questioned whether the dACC *modulates* other brain regions during tasks with simple motor control demands. Whereas this question has been studied in the context of response conflict (Fan et al., 2008), the closest precedent comes from the recent work of Schulz and colleagues (Schulz et al., 2011). With a seed in BA 32, they used the psychophysiological interaction (PPI) measure in a go-no-go task (Friston et al., 1997) to assess dACC modulation of other brain regions during response preparation. Their results provide evidence for the task-related involvement of regions of the dACC, the dPFC and the basal ganglia (but not the SMA). These investigations were conducted in adults, and whether a similar functional network structure is present in adolescence is not yet known.

Here we predicted a motor control function for the dACC in a highly simple behavioral paradigm: unimanual finger-movement in response to exogenous visual stimuli. The task itself does not contain explicit excitation or inhibition demands and (as our results show) is robustly performed by adolescents. To test involvement of the dACC in motor control, we investigated its functional interactions with the SMA, itself a prominent cortical motor region, and with M1. As is well known, M1 is the primary motor outflow region of the cerebral cortex. The SMA is hypothesized to be involved in controlling simple unimanual finger-movements that are coordinated by sensory stimuli (Romo and Schultz, 1987; Thaler et al., 1988; Picard and Strick, 2003; Grefkes et al., 2008; Witt et al., 2008). However, it is unknown whether SMA acts alone to control simple coordinated manual behavior, or whether it requires modulatory signals from other brain regions.

We investigated functional relations of the dACC with the SMA and M1 using both undirected and directed functional connectivity analysis of fMRI Blood-Oxygen-Level-Dependent (BOLD) time series data. Undirected functional connectivity analysis measures correlated fMRI BOLD activity between different brain areas (Biswal et al., 1995; Maldjian, 2001; Martuzzi et al., 2010), and was assessed for the dACC with the SMA and M1 in the task and at rest. To assess modulatory signaling from the dACC to the SMA and M1, we used directed functional connectivity analysis, which measures the predictability of fMRI BOLD activity in one brain area from that in another area by multivariate autoregression analysis (Bressler et al., 2008; Deshpande and Hu, 2012; Tang et al., 2012). By comparing directed functional connectivity in the task and at rest, we tested whether the dACC exerts *task-specific modulation* of the SMA and M1.

Our results indicate that the adolescent dACC exerts task-specific modulation of the SMA, but not M1, in visually coordinated unimanual finger movement. We suggest that this modulation reflects motor control mechanisms associated with the adolescent dACC, consistent with other studies supporting a role for the dACC in motor control (Bush et al., 1999, 2000; Ridderinkhof et al., 2004), and that its role continues into adulthood. More generally, the results directly support the concept of the dACC being crucially involved in the intentional motor control of behavior (Paus, 2001). Our focus on adolescence allows us to address the working hypothesis that the adolescent motor system evinces complex network interactions during relatively simple visuo-motor coordination.

## Materials and Methods

### Participants

Eleven (7 male, 4 female) healthy adolescent participants (age: 8–18 yrs., mean = 14 yrs.) participated in the study. The participants, all from the metro Detroit area, provided informed consent or assent under a protocol approved by the Human Investigative Committee at the Wayne State University School of Medicine, and were monetarily compensated for their participation. Participants were screened with Schedule for Affective Disorders and Schizophrenia for School-Age Children-Present and Lifetime Version (K-SADS-PL; Kaufman et al., 1997) to rule out the presence of psychiatric diagnoses. All participants were predominantly right handed as evaluated using the structured Neurological Evaluation Scale (Buchanan and Heinrichs, 1989).

### Task

Participants were instructed to tap the forefinger of their right hand as quickly as possible in response to a flashing white stimulus in the center of the display panel (RGB:255, 255, 255; extent: 34 × 32 mm; subtended visual angle: ~17º; duration: 100 ms). Four behavioral paradigms were employed, in which the stimulus was presented with frequency of either 1 Hz or 0.5 Hz, and the presentation had either Periodic or Pseudo-random intervals between stimuli. Inter-stimulus intervals (in s) for the Pseudo-random epochs (either 1 Hz or 0.5 Hz) were created by pseudo-randomly sampling values from Gaussian distributions (*μ* = 1.0 sand *σ* = 0.5 s or *μ* = 2.0 sand *σ* = 1.0 s). The lower bound on inter-stimulus intervals was 300 ms (exceeding typical lower limits in response latency). Stimulus onsets during Pseudo-random epochs were adjusted so that the average frequency of the elicited response (and therefore the number of elicited responses) was equal to the periodic counterpart. Finger responses were collected from the receptive surface (extent: 33 × 33 mm) of a fiber-optic response touchpad (Current Design Systems, Inc.) interfaced with the Presentation software package (Neurobehavioral Systems, Inc.). During the resting control condition, participants were instructed to fixate on a cross hair in the center of the field of vision. In the course of a single scan, participants alternated between epochs involving one of the four behavioral paradigms (30 s duration each, 8 epochs total) and short rest (10 s duration each, 11 epochs total). In addition, epochs 10 and 18 had long rest (30 s duration). Eight behavioral paradigm epochs (involving finger movement) formed the Task condition and 13 rest epochs (11 short and 2 long) formed the Rest condition.

### Data Collection

Functional Magnetic Resonance Imaging (fMRI) BOLD data collection was conducted on a Bruker MedSpec 4T system running the Siemens Syngo console at the Vaetkiveckius Imaging Institute in the Wayne State University School of Medicine. The scanner conformed to quality control standards exceeding clinical systems by virtue of daily quality control and SNR assessment. Whole-head gradient echo-planar images were acquired using an 8-channel head coil (TR: 2 s, TE: 30 ms, matrix: 64 × 64, 24 slices, voxels: 3.8 × 3.8 × 4.0 mm). fMRI data were collected continuously across all the conditions. An entire scan lasted 6.83 min.

### Image Preprocessing

Whole-head functional volumetric images were preprocessed with SPM5 using a standard protocol (Friston et al., 1995), including realignment to the first image in the series, correction for susceptibility-by-movement interactions, normalization to a standard EPI template, and smoothing with an 8 mm full width at half maximum (FWHM) isotropic Gaussian kernel.

In first-level analyses on individual participant data, windows of interest treated as boxcar waveforms (30 s) were convolved with the canonical hemodynamic response function (HRF) to produce reference waveforms. These waveforms were used for later contrast assessment within the General Linear Model framework. Motion effects were modeled using six movement parameters (for translation and rotation) as covariates of no interest.

### ROI Selection

Three Regions of Interest (ROIs) were considered for connectivity analysis: (1) Primary Motor Cortex, M1 (BA 4); (2) Supplementary Motor Cortex, SMA (medial BA 6) and (3) dACC (BA 24 and 32). Masks for the ROIs were generated based on the MNI atlas in the Talairach Daemon database using the WFU_PickAtlas software (ANSIR Laboratory) and applied to the whole-head functional volumetric image of each participant. As the Pickatlas mask does not specifically identify the region we label as the dACC, we identified those portions of the structure that have been most closely associated with motor control (Paus, 2001), and in separate studies have been referred to as the “midcingulate” cortex (Hoffstaedter et al., 2014, 2015). This region is also presumed to be functionally parcellated into at least two sub-divisions associated with motor function: a caudal cingulate zone and a rostral cingulate zone. The latter has been further divided into the anterior and posterior aspects, of which the latter has been more strongly associated with motor function (Milham and Banich, 2005). The dACC ROI, created in stereotactic space using BA 24 and the supra-genual aspects of BA 32 subsumed these divisions, and was anatomically distinct from, and non-overlapping with, the SMA.

Within each ROI, an effects of interest contrast was used to extract eigenvariate time series from voxels involved in the task. This contrast was based on the significance of the *F* statistic from the comparison of all task conditions, representing the weighted means of modeled effects, and was robust under the heterogeneity of the task conditions. Values were averaged over voxels within a 5 mm radial sphere (covering a volume of ~524 mm^3^) centered on the peak of the F contrast significance in each ROI. The average provides a relatively stable estimate of the representative signal around the significance peak. The sphere was located in the left hemisphere (contralateral to the finger movement behavior) for the SMA and M1, and in the hemisphere showing the higher peak of *F* contrast for the dACC. The resulting average eigenvariate intensity over a sequence of images represented the ROI time series that were then subjected to time series analysis.

### Time Series Preprocessing

Each ROI time series was first z-normalized at each time point by subtracting the mean value (over all time points) and dividing by the standard deviation (over all time points). Images with outlier intensity values were rejected. Consistent with standard practice, the first four images in the fMRI acquisition were discarded to allow signal stabilization, and did not enter in subsequent analyses. The use of motion parameters as covariates in the first level analyses removed motion effects from subsequent analyses, ensuring that our data were free of outliers from scanner inhomogeneities or motion.

### Undirected Functional Connectivity Analysis

The Pearson product-moment correlation coefficient was computed between the fMRI BOLD time series of the dACC with that of the SMA and of M1, in each participant separately for the Task (combined Periodic and Pseudo-random) and Rest conditions, and then also for the two task conditions (Periodic and Pseudo-random). All correlation values were computed using the *cor* function in the R software environment.

### Directed Functional Connectivity Analysis

Directed functional connectivity was estimated from MultiVariate AutoRegressive (MVAR) models. For both the dACC with the SMA and with M1, separate MVAR models were estimated from the fMRI BOLD time series of the ROIs for the Task (combined Periodic and Pseudo-random) and Rest conditions in each participant. The MVAR model order (Bressler and Seth, 2011), indicating the number of previous time points in the model used to estimate a current time point, was one (Tang et al., 2012).

The strength of directed functional connectivity from one ROI to another was estimated by the magnitude of a t-statistic from significance testing of the corresponding MVAR model coefficient implemented in the *glm* function in the R software environment. This strength thus served as a metric for the dynamic causal relationship between the time series, and is similar to Granger Causality in being derived from the MVAR model (Seth and Edelman, 2007; Bressler and Seth, 2011). For each participant, directed functional connectivity was estimated in both directions between the two ROIs of each model, in the Task (combined Periodic and Pseudo-random) and Rest conditions, and then also for the two task conditions (Periodic and Pseudo-random).

### Statistical Analysis, Effect Sizes, and *Post hoc* Tests

In the undirected analysis, we compared correlations between Task and Rest conditions using both the parametric paired *t*-test and the nonparametric Mann-Whitney-Wilcoxon signed-rank test. We also compared correlations between the Periodic and Pseudo-random task conditions using both the paired *t*-test and the signed-rank test. In the directed analysis, Task vs. Rest, and Periodic vs. Pseudo-random comparisons were performed using two-way, repeated-measures, within-participant Analysis of Variance (ANOVA), with Condition (Task and Rest, or Periodic and Pseudo-random) and Direction (dACC → SMA or M1, and SMA or M1 → dACC) as independent variables, and MVAR coefficient t-values as dependent variables. ANOVA was performed using the *aov* function in the R software environment. The α level for determining the significance of *F*-values was the generally accepted value of 5% (*p* < 0.05).

The effect size of a significant *F*-value for a factor or interaction was determined by computing *η*^2^ (effect sum of squares/ total sum of squares), which represents the fraction of the total variance in the dependent variable of the ANOVA that is attributable to the factor or interaction independent variable. We interpreted *η*^2^ as representing the proportion of variance in the dependent variable accounted for by a main factor or interaction (Tabachnick and Fidell, 2001).

*Post hoc* paired *t*-tests were used to analyze the results when significant main effects or interactions were found by ANOVA. The significances of these *post hoc* tests were corrected for multiple comparisons using Dunn’s multiple comparison procedure (based on the Bonferroni inequality). To control for the family-wise error rate, the alpha level for corrected *t*-values was the generally accepted value of 5% (*p* < 0.05). The effect size of a significant *t*-value was determined by computing the effect size (Pearson) correlation (r) (Cohen, 1988).

## Results

### Behavior

Response data were analyzed to assess patterns of missed responses and latencies for made responses. The percentage of missed responses and latencies for made responses were analyzed in separate repeated measures analyses of covariance with frequency and periodicity as within-participant factors, and age as covariate. No effects reached significance for the percentage of missed responses, indicating that these responses were not driven by frequency or periodicity. Separate regression analyses were used to assess age-related dependencies in the percentage of missed response. No significant relationship was observed for responses during Periodic epochs. During Pseudo-random epochs, there was a significant negative relationship between the percentage of missed responses and age, [*F*_(1,8)_ = 3.99, *p* < 0.05, one-tailed, *r*^2^ = 0.33], suggesting that the frequency of missed responses during Pseudo-random epochs decreased with age. For the analyses of latencies, there was a significant main effect of periodicity [*F*_(1,7)_ = 18.69, *p* < 0.003, *MSe* = 785.45]. Age-related dependencies assessed with regression analyses did not reveal significant effects. Response latencies during Pseudo-random epochs were on average 88 ms longer than during Periodic epochs.

### BOLD Activation

**Figure 1:**
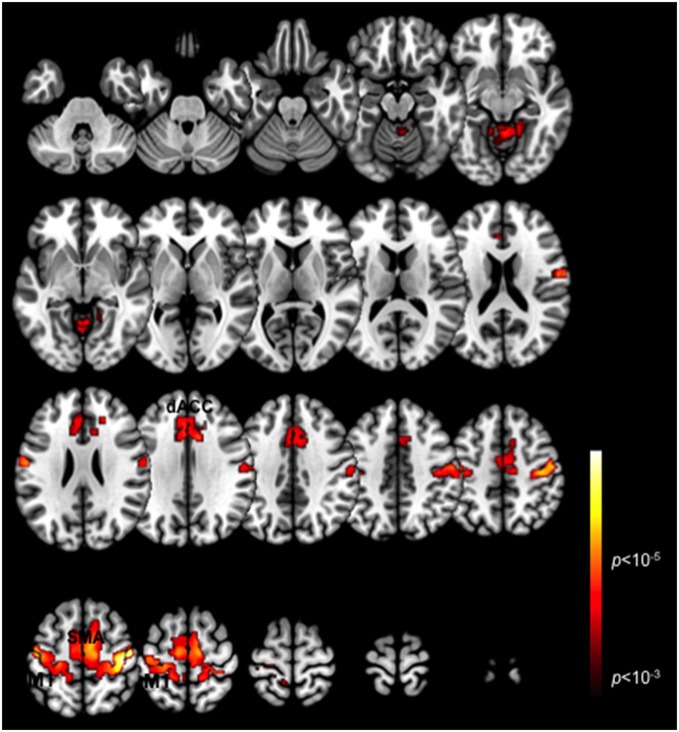
**Task-related BOLD activation maps**. Activation patterns are shown for the conjunction of the two task variants (Periodic and Pseudo-random) averaged across participants. Significant activation clusters (*p* < 0.01, cluster level) are overlaid on a mosaic of axial views that reveal task-related activations in the dACC, SMA, M1 and the cerebellum.

Figure 1 depicts a conjunction analysis (Nichols et al., 2005) performed over the Periodic and Pseudo-random epochs and thresholded in core ROIs in the motor cortex including the dACC, the SMA and M1 (*p* < 0.05, cluster level). These clusters represent overlapping patterns of activation across each of the task periods. The peak activations in the dACC converge with regions previously labeled the posterior rostral anterior cingulate (Schulz et al., 2011), and associated with motor response related activation and control. More generally these ROIs are consistent with other studies of exogenously guided finger responses (Witt et al., 2008), and are notably present in contra- and ipsi-lateral M1 consistent with other evidence of mutually supplementary activations in ipsi-lateral M1 during unimanual responses (Newton et al., 2005). The substantial overlap across the ROIs highlighted in this activation-based analysis, emphasizes the value of deriving more specific (and differentiable) network interactions driving these activations (Friston, 2005).

### Undirected Functional Connectivity

Comparisons of Task vs. Rest conditions on the undirected functional connectivity (correlation) between the dACC and the SMA were not significant by either the parametric paired *t*-test or the nonparametric Mann-Whitney-Wilcoxon signed-rank test. The same was true between the dACC and M1. By contrast, comparison of Periodic and Pseudo-random task conditions revealed that the correlations both between dACC and SMA, and between dACC and M1, were significantly greater in the Periodic than the Pseudo-random condition by both paired *t*-test [dACC and SMA: *t*_(10)_ = 3.08, *p* < 0.05 (corrected); dACC and M1: *t*_(10)_ = 3.41, *p* < 0.05 (corrected)] and by signed-rank test [dACC and SMA: *V* = 58, *p* < 0.05 (corrected); dACC and M1: *V* = 64, *p* < 0.05 (corrected)]. The effect size for dACC with SMA was *r* = 0.32. For dACC with M1, it was *r* = 0.47.

The finding of a significantly larger undirected functional connectivity between the dACC with both the SMA and M1 in the Periodic than the Pseudo-random condition suggests that the dACC activity was more tightly coupled with both these areas when the stimulus was more predictable as compared to when it was less predictable, but does not indicate the direction of influence underlying this coupling.

### Directed Functional Connectivity

To test for a significant difference between Task and Rest conditions in directed functional connectivity between the dACC and the SMA or M1, two-way Analyses of Variance (ANOVAs) were performed on the MVAR coefficient values, with Condition (Task or Rest) and Direction (dACC → SMA, SMA → dACC or dACC → M1, M1 → dACC) as factors. For the dACC with the SMA, neither the Condition nor Direction main factors were significant, but their interaction was significant [*F*_(1,10)_ = 11.28, *p* < 0.01, *η*^2^ = 0.07]. This value of *η*^2^ indicates that 7% of the total variance in the dependent variable was attributable to the interaction. For the dACC with M1, no significant main factor or interaction was observed.

**Figure 2:**
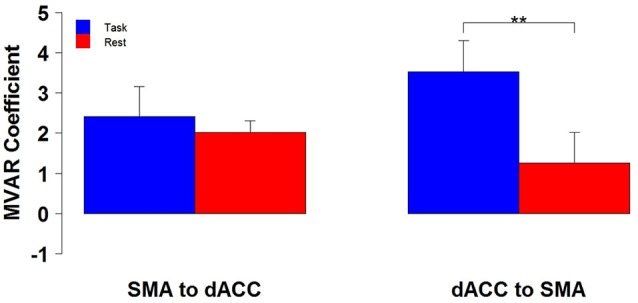
**The group mean and standard error of influence in both directions between the 2 ROIs (SMA to dACC, dACC to SMA) for Task (blue) and Rest (red) conditions**. *Post hoc* paired *t*-tests revealed that the influence from dACC to SMA was significantly greater for Task than Rest, but that the influence from SMA to dACC did not significantly differ (**p < 0.01).

**Figure 3:**
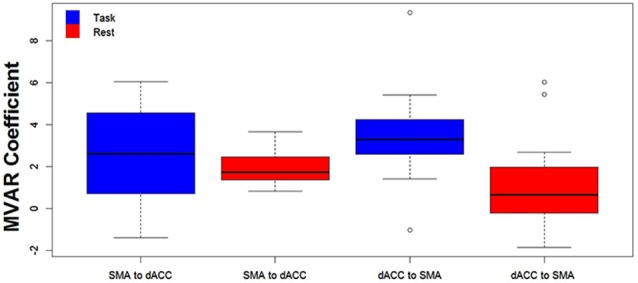
**The distribution across subjects for both directions between the 2 ROIs (SMA to dACC, dACC to SMA) and for Task (blue) and Rest (red) conditions, as box plots corresponding to the bar plots in Figure 2**. Note that the distribution for the influence from dACC to SMA during the Task is both more compact than and elevated above the distribution during Rest.

The finding of a significant interaction for the dACC with the SMA between Condition and Direction factors suggests that the difference between Task and Rest conditions significantly varied with direction. To examine this further, *post hoc* paired *t*-tests were run to compare the two conditions separately for each direction. These tests revealed that the influence from the dACC to the SMA significantly differed between Task and Rest conditions [*t*_(10)_ = 3.69, *p* < 0.01 corrected, effect size *r* = 0.41], but the influence from the SMA to the dACC did not. The effect size *r* indicates that there was a medium to strong effect between Task and Rest conditions for the influence from the dACC to the SMA (Cohen, 1988). The positive *t*-value indicates that this influence was greater for the Task than the Rest condition. This result is illustrated by the group means in Figure 2 and by the box plots in Figure 3.

**Figure 4:**
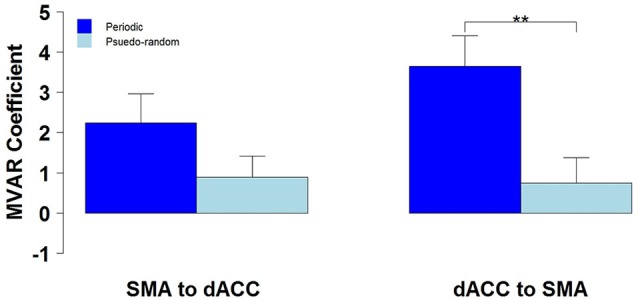
**The group mean and standard error of influence in both directions between the 2 ROIs (SMA to dACC, dACC to SMA) for Periodic (dark blue) and Pseudo-random (light blue) task conditions**. *Post hoc* paired t-tests revealed that the influence from dACC to SMA was significantly greater for Periodic than Pseudo-random conditions, but that the influence from SMA to dACC did not significantly differ (**p < 0.01).

**Figure 5:**
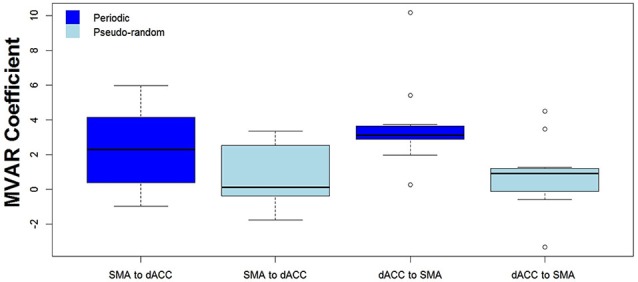
**The distribution across subjects for both directions between the 2 ROIs (SMA to dACC, dACC to SMA) and for Periodic (dark blue) and Pseudo-random (light blue) task conditions, as box plots corresponding to the bar plots in Figure 4**. Note that the distribution for the influence from dACC to SMA during the Periodic condition is both more compact than and elevated above the distribution during the Pseudo-random condition.

We then tested for a significant difference between Periodic and Pseudo-random conditions in directed functional connectivity between the dACC and the SMA, and between the dACC and M1. Again, two-way Analyses of Variance (ANOVAs) were performed, with Condition (Periodic or Pseudo-random) and Direction (dACC → SMA, SMA → dACC or dACC → M1, M1 → dACC) as factors. For the dACC with the SMA, both the Condition factor [*F* = 14.13, *p* < 0.005, *η*^2^ = 0.20] and the Condition x Direction interaction [*F* = 5.64, *p* < 0.05, *η*^2^ = 0.026] were significant. *Post hoc* tests revealed that the Periodic condition was significantly greater than the Pseudo-random condition for the influence from the dACC to the SMA [*t*_(10)_ = 4.85, *p* < 0.001, effect size *r* = 0.54], but that the difference was not significant in the reverse direction. This result is illustrated by the group means in Figure 4 and by the box plots in Figure 5.

The finding of a significant Condition factor, a significant interaction between Condition and Direction factors, and a significantly greater influence for Periodic than Pseudo-random conditions from dACC to SMA but not from SMA to dACC, suggest that the elevated undirected coupling observed between these two regions in the Periodic condition was due to the influence from dACC to SMA. The results also suggest that the observed Task vs. Rest difference in the dACC to SMA influence was due more to the Periodic than the Pseudo-random task trials.

We next used regression analysis to test whether the influence from dACC to SMA was significantly dependent on age. The *F*-value resulting from the regression analysis was not significant for either the Task or Rest condition.

For the 2-way ANOVA between the dACC and M1, the Condition factor was significant [*F* = 8.72, *p* < 0.05, *η*^2^ = 0.16], but neither the Direction factor nor the Condition x Direction interaction was significant. This finding indicates that the Periodic condition was significantly different from the Pseudo-random condition, regardless of direction. In fact, the mean influence, regardless of direction, was greater for the Periodic than Pseudo-random condition (Figures 4, 5). This finding that the directional functional connectivity between dACC and M1 was greater in the Periodic than the Pseudo-random condition, regardless of direction, is consistent with that from the undirected functional connectivity between dACC and M1. It suggests that the Direction factor was not important for the functional relation between these two regions.

## Discussion

In this study, we used time series analysis to investigate undirected and directed functional interactions of the adolescent dACC with the Supplementary Motor Area (SMA) and the Primary Motor Cortex (M1) during a simple unimanual visuomotor task. Undirected functional connectivity analysis revealed significantly greater coupling of dACC with both SMA and M1 for a proactive than a reactive task condition. Directed functional connectivity analysis demonstrated a significant task-specific modulatory influence from the dACC to the SMA, but not in the reverse direction, and in neither direction between the dACC and M1. Although our results were obtained in adolescents, they motivate the search for comparable analyses in adults. The employed behavioral paradigm is a very basic sensorimotor coordination task well performed by the adolescent subjects and un-confounded by development effects on task-proficiency.

The participants in this study were healthy adolescents. Even though *cognitive control* remains dynamic across adolescence, basic mechanisms of sensorimotor integration are relatively mature by then (Witt and Stevens, 2013). We demonstrate that the task-specific modulatory influence from the dACC to the SMA was independent of age, suggesting that motor-control-related network signaling by the dACC during simple tasks is sophisticated in adolescence. The maturing of dACC-related motor control thus may occur before maturation of higher order cognitive control. Undoubtedly further testing will be required in the future with adults performing the same task (and this is a focus of our ongoing studies).

Our analyses demonstrate a task-specific directed functional relation from the dACC to the SMA in unimanual visuomotor control, thus suggesting a specialized role for the dACC in human motor control. Although it is generally accepted that executive control depends on the coordination of sensory and motor operations by frontal regions of the brain (Funahashi, 2001; Miller and Cohen, 2001), different frontal regions are responsible for different aspects of executive control.

The finding that the dACC→SMA influence was significantly larger for the Periodic than Pseudo-random task conditions suggests that the strength of this influence is greater when the subject is in a proactive (Periodic) as compared to a reactive (Pseudo-random) state. This difference may reflect the maintained expectancy of stimulus timing that is inherent to proactive states. Maintaining the system in a proactive, as opposed to a reactive response state, presumably requires ongoing adjustments in control. Control and behavioral adjustments, particularly during conflict robustly activate the anterior cingulate cortex (Botvinick et al., 1999; Kerns et al., 2004; Sohn et al., 2007; Fan et al., 2008). Whereas the specific demands induced by response conflict differ from the demands of maintaining a response set during finger tapping, we surmise that our results reveal that the dACC is involved in the continual fine tuning of finger responses during a proactive response set. This result is an extension of previous work (Kerns et al., 2004), while providing evidence of network profiles of the dACC to the SMA.

Our results provide a measure of continuity with the work of Grefkes and colleagues (Grefkes et al., 2008). Using a task with manual (left or right) or bimanual responses to a flashing circle (1.5 Hz), they employed dynamic causal modeling (DCM) to assess connectivity and dynamics in a sub-network involving bilateral SMA, M1 and pre-motor cortex. They showed that the intra hemispheric SMA → M1 pathway was positively modulated when responding with the contra-lateral response hand, whereas the bilateral SMA → M1 pathways were modulated during bimanual responses. Although the dACC was not included in their network analyses, their results suggest a hierarchical organization within the motor system. By comparison, our results (though achieved using a complementary analytic method) suggest that the dACC, a region that is associated with more general mechanisms of cognitive control, exercises substantial modulation of the SMA as a function of motor task demand. These observations emphasize the value of effective connectivity techniques in revealing the complex and “causal” dynamics of the motor system.

By employing both undirected and directed functional connectivity, our study demonstrates the relative value of these two types of measure. Using undirected functional connectivity analysis, elevated coupling was observed between dACC and both SMA and M1 in the proactive, as compared to the reactive, condition. Directed functional connectivity analysis was required to disambiguate the pattern of directed interactions underlying the undirected functional coupling: that between dACC and SMA was supported by a unidirectional influence from dACC to SMA, whereas that between dACC and M1 was supported by bidirectional influences between the two areas.

The statistical bases of time series analyses do not allow us to determine the anatomical pathway over which modulatory signals might be conveyed between areas. This, and other limitations associated with the BOLD signal, means that we are unable to determine whether the presumed unidirectional modulation of the SMA by the dACC is monosynaptic or polysynaptic. Nonetheless, our findings are consistent with other evidence that the dACC provides supplementary functionality to the SMA (Hatanaka et al., 2003).

The application of autoregressive time series analysis to fMRI BOLD data has been controversial because of the possibility of inter-regional variation in the HRF (David et al., 2008; Friston, 2009, 2011; Roebroeck et al., 2011a,b; Smith et al., 2011; Valdes-Sosa et al., 2011; Stephan and Roebroeck, 2012; Friston et al., 2013), or measurement noise (Seth et al., 2013). However, neither of these potential problems can account for the dependence of the dACC-to-SMA influence on experimental condition in our analyses, since neither HRF nor measurement noise is expected to differ with experimental condition.

The observation that only the directional influence from the dACC to the SMA significantly distinguished task from rest suggests that the dACC may reside at a hierarchically higher organizational level than the SMA in motor control. Since the SMA is a premotor region, this result supports a triune cortical hierarchy of frontal executive networks (Fuster, 2003). In this view, the dACC is hierarchically above the SMA, and selectively modulates it as part of the SMA’s function in controlling simple coordinated manual behavior.

Our results inextricably link the dACC with the motor system, suggesting that the SMA is not alone in driving motor behavior. They open the possibility for differential contributions by the dACC and the SMA to motor behavior, such that the dACC might provide high-level control of the SMA. However, our analyses do not preclude the possibility of other “higher-order” areas being involved. For example, to maintain visually coordinated motor behavior in the present task, the dACC would need to be informed by the visual system. Our analyses did not allow us to infer whether the dACC receives direct projections from visual cortex (Sepulcre, 2014), or whether visual information is first passed to another “higher-order” area, which then informs the dACC. More detailed experimentation is needed to determine all the areas involved in motor control and the various conditions of their involvement.

## Conclusions

We recorded and analyzed fMRI BOLD data from healthy adolescent participants performing a visually coordinated unimanual finger-movement task. The observation of task-specific modulatory signals to the SMA from the dACC indicates that the dACC is systematically involved in motor control in adolescence, consistent with its predicted role in motor control. Adolescent neuro- and behavioral-development is heterochronous, and brain interactions in the adolescent state are not necessarily predictive of the adult state. Nevertheless, the lack of age-related effects in our analyses suggests that both the behavioral domain, and the functional brain responses it evoked were invariant. It is plausible, though as yet unknown, that the combination of task and time series analyses we applied will reveal similar principles of motor organization in adults. We suggest that control mechanisms in core and extended motor circuitry may be hierarchically organized, with the dACC operating at a higher hierarchical level of motor control than the SMA.

## Conflict of Interest Statement

The authors declare that the research was conducted in the absence of any commercial or financial relationships that could be construed as a potential conflict of interest.
